# Phylophenetic properties of metabolic pathway topologies as revealed by global analysis

**DOI:** 10.1186/1471-2105-7-252

**Published:** 2006-05-09

**Authors:** Yong Zhang, Shaojuan Li, Geir Skogerbø, Zhihua Zhang, Xiaopeng Zhu, Zefeng Zhang, Shiwei Sun, Hongchao Lu, Baochen Shi, Runsheng Chen

**Affiliations:** 1 Bioinformatics Laboratory and National Laboratory of Bromacromolecules, Institute of Biophysics, Chinese Academy of Sciences, Beijing 100101, China; 2 Bioinformatics Research Group, Key Laboratory of Intelligent Information Processing, Institute of Computing Technology, Beijing 100080, China; 3 Graduate School of the Chinese Academy of Sciences, Beijing, China

## Abstract

**Background:**

As phenotypic features derived from heritable characters, the topologies of metabolic pathways contain both phylogenetic and phenetic components. In the post-genomic era, it is possible to measure the "phylophenetic" contents of different pathways topologies from a global perspective.

**Results:**

We reconstructed phylophenetic trees for all available metabolic pathways based on topological similarities, and compared them to the corresponding 16S rRNA-based trees. Similarity values for each pair of trees ranged from 0.044 to 0.297. Using the quartet method, single pathways trees were merged into a comprehensive tree containing information from a large part of the entire metabolic networks. This tree showed considerably higher similarity (0.386) to the corresponding 16S rRNA-based tree than any tree based on a single pathway, but was, on the other hand, sufficiently distinct to preserve unique phylogenetic information not reflected by the 16S rRNA tree.

**Conclusion:**

We observed that the topology of different metabolic pathways provided different phylogenetic and phenetic information, depicting the compromise between phylogenetic information and varying evolutionary pressures forming metabolic pathway topologies in different organisms. The phylogenetic information content of the comprehensive tree is substantially higher than that of any tree based on a single pathway, which also gave clues to constraints working on the topology of the global metabolic networks, information that is only partly reflected by the topologies of individual metabolic pathways.

## Background

Genes usually do not act individually but form functional or structure organizations, exemplified by metabolic pathways. As metabolic pathways are essential to the survival of organisms, and their evolution has been under debate for more than half a century [1], a combined phylogenetic and phenetic analysis of pathway topology might expand the understanding of the evolutionary processes molding their form and structure.

Up to now, more than 280 organisms have been fully sequenced, and the Kyoto encyclopedia of genes and genomes (KEGG: [2]) has computationally reconstructed organism-specific pathways based on genome information and reference pathways, thereby making it possible to compare metabolic pathways or networks between species. Several groups have carried out phylogenetic analyses based on metabolic pathways, deriving phylogenetic trees from the information of individual pathways [3-5], the presence and absence of entire pathways [6], or the reaction content of entire pathways [7]. Other groups have reconstructed the phylogenies based on the enzyme content of entire metabolic networks [8,9]. One of these groups used a method based on the combination of sequence information and graph topology [3,4]. The combination of these two sources of information was also used for analyzing protein-protein interaction networks, known as PathBLAST [10,11]. These studies have provided valuable insight into the evolution of metabolism; however, as phylogenetic trees they have generally diverged substantially from trees based on 16S rRNA, the most used molecule for phylogeny reconstruction. A common feature of phylogenetic trees based on metabolic information is that, owing to similar evolutionary pressures, organisms in similar habitats tend to be clustered together, and Aguilar *et al*. [9] therefore regarded such trees as phenetic rather than phylogenetic. Furthermore, one group showed that trees based on different subsets of metabolic networks were different [9], and another result also indicated a similar situation when several different pathways were used to construct trees separately [5], just like when different molecules are used for reconstructing phylogenies [12].

Our study extends previous works in two ways. First, in order to elucidate to what extent the topologies of single pathways, as functional elements of an organism, provide phylogenetic information and reflect evolutionary pressures, we have subjected information on all available metabolic pathways to topological analysis. Second, we have attempted to measure the phylogenetic information content of larger pathways sets, or partial metabolic networks, compared to single metabolic pathways. Considering that the topologies of metabolic pathways are phenotypic features derived from heritable characters, they will necessarily contain both phylogenetic and phenetic components, and we have therefore regarded the properties derived from pathway topologies as "phylophenetic". The work has proceeded through three steps. First, a topologically based definition for metabolic pathway profiles was introduced; hence the taxonomic distributions of pathways were studied. Second, for each metabolic pathway, a phylogeny based on the topological similarity was reconstructed and quantitatively compared to the corresponding 16S rRNA-based tree. Finally, by using the quartet method, the trees based on single pathways were combined into one comprehensive tree, which showed a much higher similarity to the corresponding 16S rRNA-based tree than any tree based on a single pathway.

## Results and discussion

### Taxonomiac distributions of pathways

**Figure 1:**
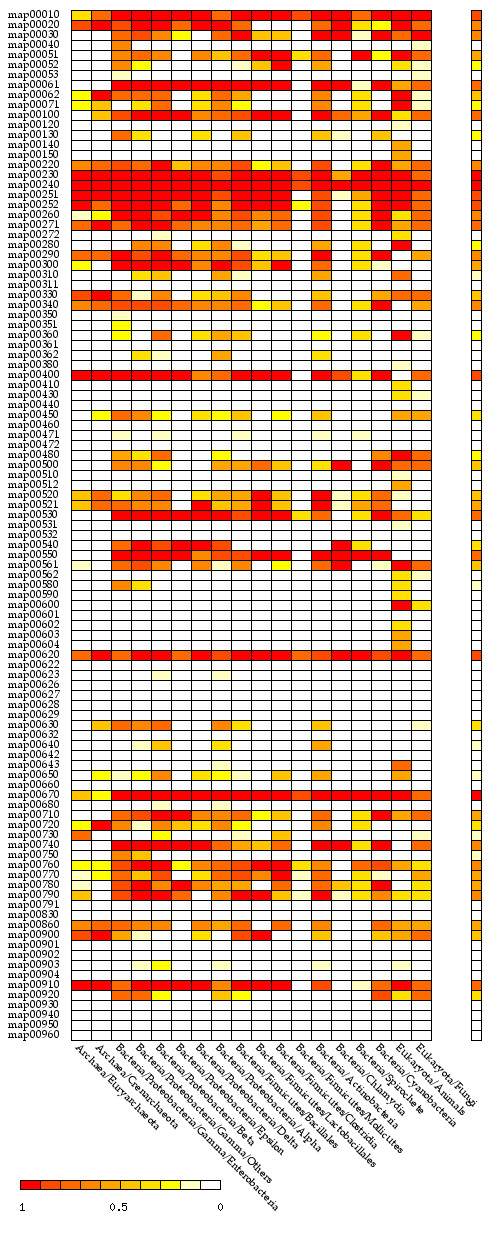
**The distribution of pathway specific organisms in different phylogenetic categories. **The grid color indicates the fraction of organisms in each category containing a certain pathway. The definition of presence or absence of metabolic pathways is given in Methods.

**Figure 2:**
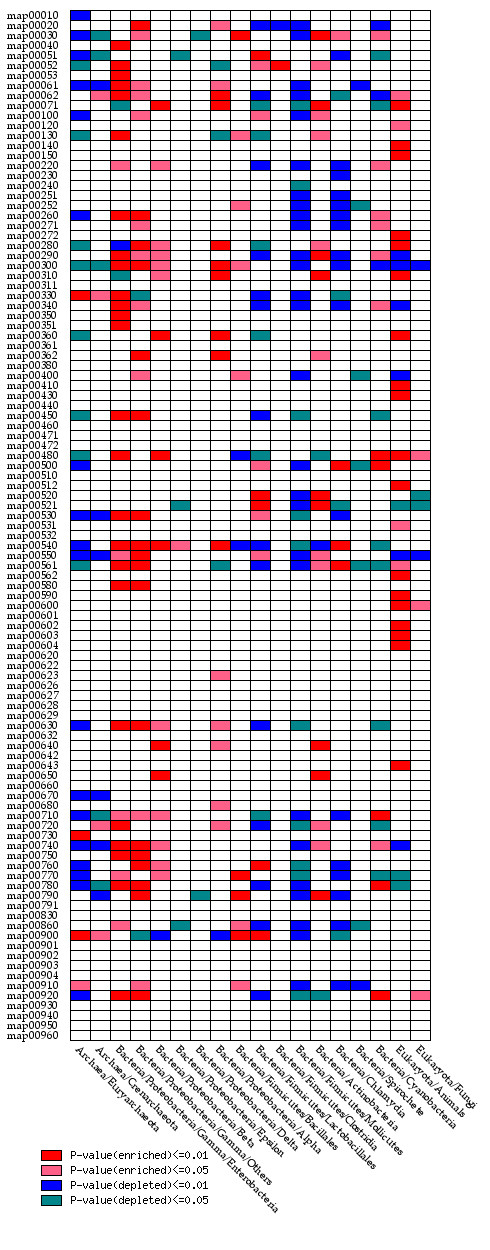
**The distribution of P-values in differentphylogenetic categories. **The P_enriched_-values and P_depleted_-values were calculated by using the equation 1 and equation 2 (see Methods), measuring whether a pathway specific subset was enriched in or depleted of organisms from a particular category (only P-values less than 0.05 are shown).

In order to obtain an overview of the distribution of pathways relative to the phylogenetic classification of the 184 organisms studied, we produced a grid showing the fraction of organisms in each KEGG [2] category containing a given pathway, based on the definition given in Methods (Figure 1). Organisms containing any given pathway were far from equally distributed among the phylogenetic categories. Therefore, using equations 1 and 2 separately, pathway specific P-values were assigned to measure whether a pathway specific subset was enriched in or depleted of organisms from a particular category (Figure 2). For pathways found in only a small number of studied organisms, these organisms usually belonged to one or two phylogenetic categories. For example, the C21-steroid hormone pathway (map00140) was present only in three of the organisms studied, all animals (P_enriched_-value = 9.8 × 10^-6^). On the other hand, for a pathway conserved in most organisms, there were usually some phylogenetic categories in which none or only a few organisms contained the given pathway. For example, the riboflavin metabolism (map00740) was present in 126 of the 184 organisms, while only 1 of 19 archaea contained it (P_depleted_-value = 1.7 × 10^-9^), which is consistent with previous findings that several steps in the biosynthesis of riboflavin in archaea were absent [13]. In addition, many metabolic pathways were absent in obligate parasites like Chlamydia and Mollicutes.

### Phylophenetic properties of single pathways

**Figure 3:**
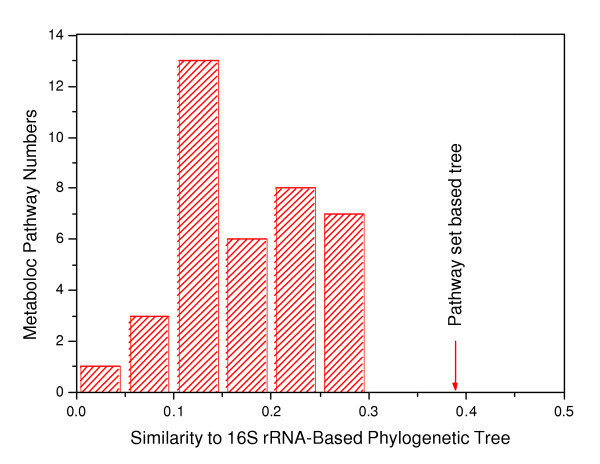
**The distribution of similarity values betweensingle pathway-based trees and 16S rRNA-based trees. **Thirty-seven pathways were studied. The similarity value between the pathway set based tree and the corresponding16S rRNA-based tree is indicated by an arrow.

To be able to compare to 16S rRNA-based trees, for each pathway that was present in more than 10 prokaryotes, a phylophenetic tree was reconstructed based on the pathway topology for the pathway specific subset of prokaryotes (details shown in Methods, with distance definition in equation 3). As little evolutionary information could be acquired from the most conserved pathways, only metabolic pathways whose topological variation between organisms is significant enough were studied. A total of 37 pathway specific phylophenetic trees were inferred and compared to 16S rRNA-based trees for the corresponding prokaryotes. The similarity values for each pair of trees (single pathway-based and 16S rRNA-based trees) ranged from 0.044 (nucleotide sugars metabolism, map00520) to 0.297 (valine, leucine and isoleucine degradation, map00280), with nearly 90% (33 out of 37) of the pathways having a similarity value of more than 0.1 (Figure 3; Table 1). For each of the 37 pathways, 1,000 random trees for the same subset of organisms were produced. Only 0.23% (86 out of 37,000) of the random trees had similarity values (compared to 16S rRNA-based trees) of more than 0.1, showing that topological variations in metabolic pathways contain a certain measure of phylogenetic information. When using Heyman and Singh's distance definition [5], similar results were obtained, also with nearly 90% (32 out of 37) pathways having similarity values above 0.1 (Table 1). The consistency in the results indicates that these two topology-based distance definitions are equally good at preserving evolutionary information. The result is similar to a previous analysis of the topology of the citric acid cycle (map00020) and glycolysis/glucogenesis (map00010), where the clustering of organisms agreed well with the NCBI taxonomy [5]. Comparisons of trees based on glycolysis/glucogenesis to the NCBI taxonomy gave similarity values of 0.18 and 0.19 for two sets of 48 and 72 organisms, respectively [5], very close to the value of 0.178 calculated by our method for 154 organisms containing this pathway. As the similarity values of these two pathways are only intermediate among all the 37 studied pathways, this should indicate that most single pathways contain a substantial amount of phylogenetic information.

**Figure 4:**
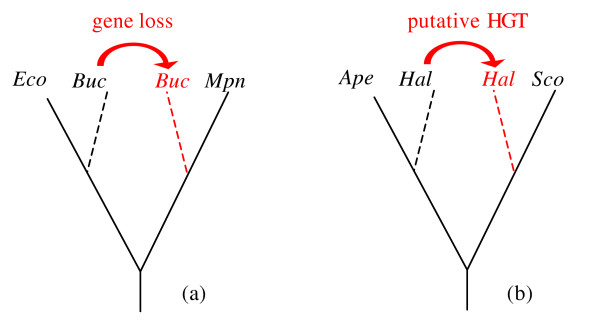
**The differences between single pathway-based treesand 16S rRNA-based trees. **(a) In the 16S rRNA-based tree, two γ-proteobacteria *Buchnera aphidicola *(Buc) and *Escherichia coli *(Eco) are clustered together. In the phylophenetic tree based on the topology of the glycolysis/gluconeogenesis (map00010), the obligate intracellular symbiont *B. aphidicola *(marked with red color) is clustered with the obligate intracellular parasite *M. pneumoniae *(Mpn), mainly due to the gene loss in the two organisms. (b) In the 16S rRNA-based tree, two archaea *Halobacterium sp. NRC-1 *(Hal) and *Aeropyrum pernix *(Ape) are clustered together. In the phylophenetic tree based on the topology of the selenoamino acid metabolism (map00450), *Halobacterium *(marked with red color) is clustered with actinobacteria *Streptomyces coelicolor *(Sco), mainly due to the putative horizontal gene transfer of selenocysteine lyase.

The trees derived from topological information of each of the 37 single pathways generally had a different information content than those derived from 16S rRNA. Whereas the main phylogenetic aspects of evolution are conserved to some extent, these trees also portray how the essentiality of specific metabolic pathways has shaped the evolutionary paths of different organisms. Thus, different aspects of evolutionary pressure result in the different similarity values when comparing to 16S rRNA-based trees, because, as functional features, the operation of metabolic pathways is essential to the survival of organisms; and due to selection on these feature, the topologies of the same pathways in two organisms with short divergence distance might be quite different if the selection pressures differs. For example, although the divergence distance between two γ-proteobacteria *Buchnera aphidicola *(Buc) and *Escherichia coli *(Eco) is much shorter than that between *B. aphidicola *and the mollicute *Mycoplasma pneumoniae *(Mpn), due to their similar environments, the topology of the glycolysis/gluconeogenesis (map00010) for the obligate intracellular symbiont *B. aphidicola *is much more similar to that of the obligate intracellular parasite *M. pneumoniae *(Figure 4a). Another example is the phylogenetically closely related archaea *Halobacterium sp. NRC-1 *(Hal) and *Aeropyrum pernix *(Ape). In the tree based on the selenoamino acid metabolism (map00450), *Halobacterium *is clustered with an actinobacteria *Streptomyces coelicolor *(Sco), rather than with *A. pernix*, mainly due to the influence on pathway topology from selenocysteine lyase (EC 4.4.1.16). This enzyme has been found in more than 91 studied bacteria, but among archaea only in *Halobacterium*, most likely due to horizontal gene transfer (HGT) (Figure 4.b). Thus, pathway topologies can be regarded as the results of a compromise between phylogenetic information inherited from the last common ancestor and evolutionary pressure causing more rapid shifts in metabolic structure, and varying similarity values may reflect the strength of the two factors.

Calculation of various topological parameters for each of the 37 pathways (Table 1) showed that except for vertex number, no obvious correlation could be found between the similarity values and other topological parameters, including diameter, average path length and clustering coefficient of the reference pathway graphs. Nor did the similarity values display any obvious correlation to pathway category or number of pathway specific prokaryotes. However, with increasing number of vertices in a graph (i.e. enzymes in a pathway), the pathway-inferred trees had a tendency towards higher similarity to the corresponding 16S rRNA-based tree. When considering a model of complex networks, if one new vertex is added, the number of all possible sub-networks is doubled. Therefore, the amount of potential variation within a pathways graph could be expected to increase exponentially in relation to vertex number. As the opposite is observed (i.e. a conservation of phylogenetic information with increasing vertex number), this implies that most of the potential topological variation in metabolic pathways is never realized, possibly due to strong evolutionary constraints on topological variation, particularly in larger pathways.

The topologies of the remaining 66 pathways provided little phylophenetic information, mainly for two reasons. One was that 47 of the pathways were either only found in a small number of specialized organisms, or were too incomplete to be regarded as present in most studied organisms. For example, although glycosphingolipid metabolism (map00600) widely exists in animals, its topology provided little evolutionary information as only 5 animals were available for study; given more animal genomes and proper methods, it should be possible to derive evolutionary information also from this pathway. The other reason was that the remaining 19 pathways were too conserved to contain any useful information for distinguishing between organisms. For example, the peptidoglycan biosynthesis (map00550) exists in 135 prokaryotes; however specific pathway graphs for 101 of these were identical, and it was therefore impossible to derive any relationships between these organisms based on the topological information.

### Phylophenetic properties of the pathway set based tree

**Figure 5:**
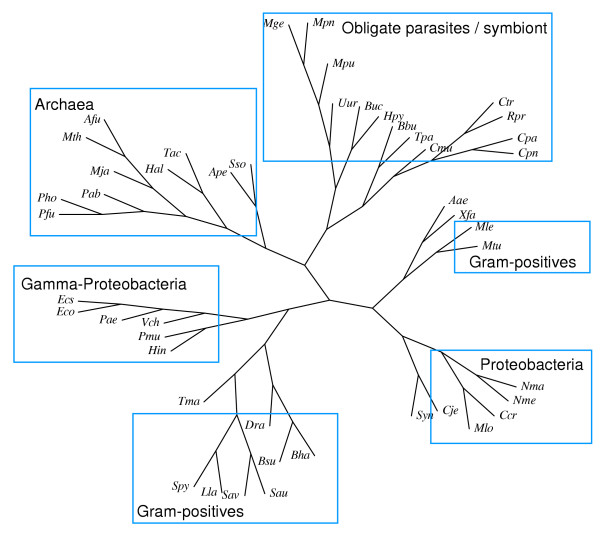
**The pathway set based tree. **The names and categories of organisms included in the tree are given in Additional file 1. The tree was drawn with the program TreeconW [35].

Based on the topologies of the 37 single pathway trees, a comprehensive tree was constructed using the quartet method described in Methods (Figure 5). In order to compare this pathway set based tree to trees based on 16S rRNA, gene content and gene order, we limited tree construction to 47 organisms for which relevant data was available in the SHOT server [14]. Comparison of the pathway set based tree to the 16S rRNA-based tree gave a similarity value of 0.386, which was higher than for any single pathway (Figure 3). As single pathways only provided topological information of "branches" to reconstruct the pathway set based tree, the higher similarity value for the pathway set based tree might indicate the existence of global constraints working on the global metabolic networks topology, which are reflected by the individual metabolic pathways. However, with the exception of the gene order tree based on SHOT version 2.0, the other three gene order and gene content based trees showed higher similarity to 16S rRNA-based tree than the pathway set based tree (similarity values ranging from 0.522 to 0.613; Table 2), indicating that evolutionary influences generally not accounted for by the rRNA-based phylogeny (e.g. HGT, gene loss and evolutionary pressures) have made a stronger mark on the topology of the metabolic networks than on the genome as a whole.

The pathway set based tree reflected both the classical taxa and the living styles of analyzed organisms (Figure 5). It showed a definite separation between archaea and bacteria, with all 10 archaea clustered in the same branch and the two-crenarchaeotan species (*Sulfolobus solfataricus *(Sso) and *A. pernix *(Ape)) being separated from other 8 euryarchaeota on two different sub-branches. Among the eubacteria, the largest branch contained 13 organisms which all belonged to obligate parasites or obligate symbionts with small genomes. This clustering of intracellular parasites/symbionts from various rRNA based taxa is a persistent feature of metabolism-based phylogenies [7-9], and reflects a convergence towards small metabolic networks consisting of functions that cannot be substituted by import of host-produced metabolites. Of the 8 γ-proteobacteria, 6 were placed in one branch; the two exceptions were *B. aphidicola *(Buc), an obligate intracellular symbiont which clustered with other obligate parasites or symbionts, and *Xylella fastidiosa *(Xfa), the only plant pathogen. Interestingly, the latter shared a separate branch with two other pathogens (*Mycobacterium lepra *(Mle) and *M. tuberculosis *(Mtu)), possibly indicating some relatedness in metabolic design also for extracellular pathogens. The fourth member on this branch was the hyperthermophilic *Aquifex aeolicus *(Aae), which also cluster close to *X. fastidiosa *based on enzyme content [8], and relatively close to the mycobacteria in the 16S rRNA tree. Another branch included 6 organisms, which, with the exception of the cyanobacterium *Synechocystis sp*. (Syn), all belonged to non-γ proteobacteria, whereas an additional two parasitic non-γ subclass proteobacteria (*Rickettsia prowazekii *(Rpr) and *Helicobacter pylori *(Hpy)) were grouped with the obligate intracellular parasites/symbionts. The nine Gram-positive organisms were placed in two branches; two mycobacteria were clustered together in one branch, and the remaining seven in another branch also containing the hyperthermophilic organism *Thermotoga maritima *(TMA). *T. maritima *is much of an orphan in bacterial phylogeny. Whereas the 16S rRNA-based tree places it on a branch together with the other hyperthermophilic *A. aeolicus*, it ends up on a separate branch on a gene content tree [15], and in close proximity to the bacillales in the metabolic reaction content tree [7].

The phylogenetically most interesting aspect of the pathway set based tree is that it coincides with the consensus genome tree [15] in clustering chlamydiae and spirochetes among the bacteria, and methanogen and pyrococci among the archaea. Both of these two 'new clades' are strongly supported by information derived from entire genome information, but not by rRNA phylogenies. This strongly indicates that the combination of metabolic pathway topologies not only depicts phenotypic similarities between different groups of prokaryotes, but also contains a substantial measure of phylogenetic information.

### Evaluation of the approach

As one of the types of complex intracellular networks, metabolic networks have drawn much attention in recent years [16-18]. Our results relate to two aspects of complex networks. First, our results show that the topological variations of metabolic pathways can reflect the adaptation to specific evolutionary pressures, indicating the possible relationships between the structure of a metabolic pathway (which can be regarded as a type of functional module) and organismal adaptation. Secondly, the results touch upon the relation between phylophenetic properties at different levels of the metabolic networks. One of these levels refers to the metabolic pathway, or functional module, the other refers to the sets of pathways, or the partial metabolic networks. The study of evolutionary relations between different levels of networks will not be limited to metabolic networks, but may also be applicable to other types of networks like transcriptional regulation networks and protein-protein interaction networks. However, as this study mainly concentrated on the topological information of metabolic pathways, it will have some limitations. Whereas our study gave insight into evolutionary pressures that might shape the topology of metabolic pathways, it could not show how the modified pathway might help the organism adapt to a specific environment. In further studies, more biochemical analysis, such as flux balance analysis [19], could be used to perform more detailed studies on the relationships between pathway topology and evolutionary pressures.

**Figure 6:**
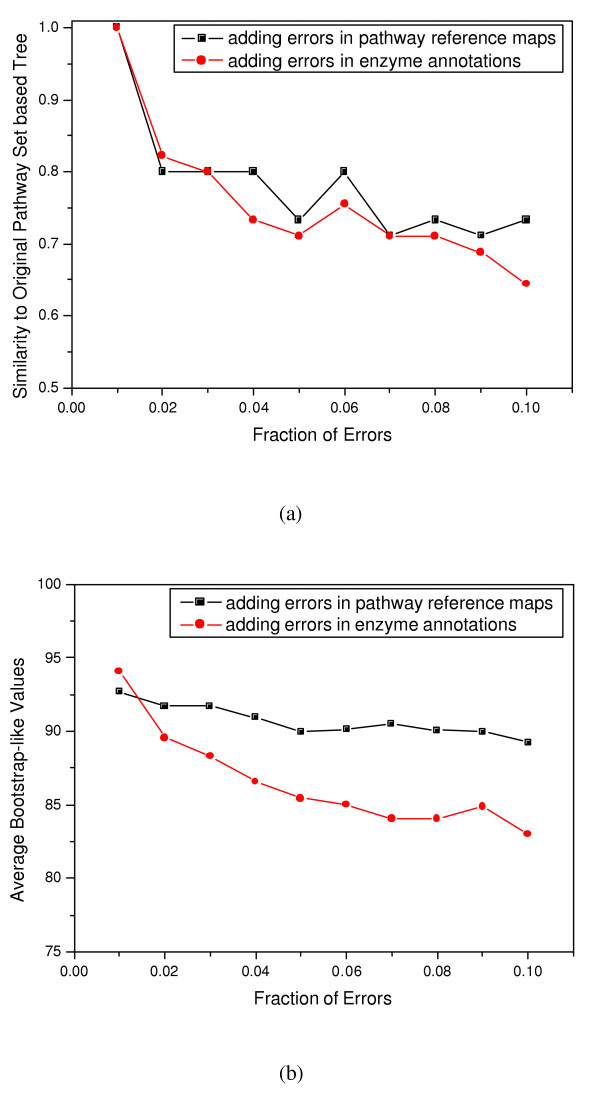
**Robustness testing. **(a) Similarities between consensus pathway set based trees and the original one. (b) Average bootstrap-like values of consensus pathway set based trees.

Though the KEGG pathway reference maps and KEGG enzyme annotations are probably among the most reliable available datasets of this kind, errors in reference maps or annotation may occur, and could affect our results. To test the robustness against such errors we randomly added specified errors (from 1% to 10%, with 1% intervals) in either the reference graph of each metabolic pathway, or in the KEGG annotation of enzymes for each organism. For each interval we repeated the whole analysis 100 times, thereby acquiring a consensus pathway set based tree with bootstrap-like values (see details in Methods). To obtain a measure of the robustness, two sets of values for consensus pathway set based trees were calculated: the similarity to the original tree, and the average of bootstrap-like values of all branches. The former reflects the variation of pathway set based tree given a specific error and the latter the stability of pathway set based tree against specific errors. Both values decreased when the fraction of added errors increased (Figure 6). However, even with an error rate up to 10% most features of original pathway set based tree were preserved; e.g. both the branch of obligate parasites/symbionts and the branch of archaea were persistent up to 10% error rate. The test indicates that a moderate amount of errors in KEGG pathway maps or enzyme annotations will not influence our results substantially.

## Conclusion

This analysis has clearly shown that the topologies of different metabolic pathways contain different phylogenetic and phenetic information content. This suggests that pathway topologies can be regarded as the results of a compromise between phylogenetic information inherited from the last common ancestor and evolutionary pressure causing more rapid shifts in metabolic structure, and varying similarity values might reflect the strength of the two factors. The analysis has also shown that the phylogenetic information content of the pathway set based tree is substantially higher than that of any tree based on a single pathway, which indicates that metabolic pathway evolution might be influenced by the potential constraints working on the topology of global metabolic networks. When more organisms, especially eukarya, are available in KEGG, the details of the compromise between phylogenetic information and evolutionary pressure on metabolism features as well as global constraint working on metabolic networks topology might be more thoroughly studied.

## Methods

### Deriving specific pathway graphs for each organism

Two complementary ways of representing metabolic pathways or networks have commonly been used for topological based analysis [5,16,20-23], using either enzymes or metabolites as vertices. In this work, for each reference pathway in KEGG PATHWAY [2], a corresponding reference graph was obtained by using enzymes as vertices. The enzymes were denoted by the Enzyme Classification (EC) number, and a directed edge leading from an enzyme E1 to an enzyme E2 arise if the compound A is a product of a reaction catalyzed by E1 and a substrate of E2. Totally, 103 reference graphs were manually retrieved.

A total of 184 fully sequenced organisms were used in this work, including 19 archaea, 152 bacteria and 13 eukarya; all being annotated in KEGG GENE [2]. The phylogenetic categories of the selected organisms were also obtained from KEGG, which classified the organisms into 3 domains and 27 categories (Additional file 1). The information of whether or not a specific enzyme is present in a specific organism is available in the ENZYME section of the KEGG LIGAND [2]. By combining the information on enzyme presence with reference graphs, and removing isolated vertices, specific pathway graphs for each organism were derived. Therefore, for each metabolic pathway, any organism-specific graph is a sub-graph of the reference pathway graph.

### Definition of presence or absence of pathways

We introduced a new topology-based definition for whether a pathway exists in a given organism. If the diameter of an organism-specific pathway graph was larger than the average path length of the reference pathway graph, the pathway was regarded as present in the organism, if shorter, the pathway was regarded as absent. The average path length of a directed pathway graph was calculated as the average number of edges in the shortest path between any pairs of reachable vertices. The diameter of the graph was the maximum shortest path length between any two reachable vertices.

There are several possible definitions that could be used to determine whether or not a pathway is present in an organism. For example, Liao *et al*. [6] used a strict definition for whether a pathway from the WIT database [24] existed in a given organism, requiring that all enzymes of the WIT pathway be present. This definition is too strict for this work, as most reference pathways in KEGG are quite complex. Other candidate definitions include requiring that a specific fraction (such as 1/3, 1/2 or 2/3) of enzymes in one pathway (or edges in one pathway graph) are present in an organism. Those definitions do, however, not consider topological information, whereas our new definition focuses on the continuity of chemical reactions, represented by the diameter of an organism-specific pathway graph. We compare the diameter of an organism-specific pathway graph to the average path length of the reference pathway graph, which is one of the most important topological parameters of a graph and also taken as the characteristic path length of the graph [25]. The definition is effective because if a pathway is defined as present in an organism, the pathway graph in that specific organism must contain at least one series of continuous chemical reactions with relative large path compared to the size of reference graph, and, on the other hand, if a pathway is defined as absent in an organism, it would at least be very incomplete or very unconnected.

### Assignment of pathway specific P-values to categories

Given the topological definition of absence and presence of pathways, for each metabolic pathway there was a pathway specific subset of organisms that contained it. Usually, the organisms in a pathway specific subset were not equally distributed among the different phylogenetic categories. P-values [26,27] were therefore calculated to distinguish categories that tended to be significantly enriched or depleted in each pathway specific subset (categories containing less than 3 organisms were not included).

Hypergeometric distribution was applied to model the probability of observing by chance, at least k organisms in a pathway specific subset size n belonging to a category containing C organisms from the total number of G organisms, such that the P_enriched_-value was given by

Penriched=1−∑i=0k−1(Ci)(G−Cn−i)(Gn).     (1)
 MathType@MTEF@5@5@+=feaafiart1ev1aaatCvAUfKttLearuWrP9MDH5MBPbIqV92AaeXatLxBI9gBaebbnrfifHhDYfgasaacH8akY=wiFfYdH8Gipec8Eeeu0xXdbba9frFj0=OqFfea0dXdd9vqai=hGuQ8kuc9pgc9s8qqaq=dirpe0xb9q8qiLsFr0=vr0=vr0dc8meaabaqaciaacaGaaeqabaqabeGadaaakeaacqWGqbaudaWgaaWcbaGaemyzauMaemOBa4MaemOCaiNaemyAaKMaem4yamMaemiAaGMaemyzauMaemizaqgabeaakiabg2da9iabigdaXiabgkHiTmaaqahabaWaaSaaaeaadaqadaqaauaabeqaceaaaeaacqWGdbWqaeaacqWGPbqAaaaacaGLOaGaayzkaaWaaeWaaeaafaqabeGabaaabaGaem4raCKaeyOeI0Iaem4qameabaGaemOBa4MaeyOeI0IaemyAaKgaaaGaayjkaiaawMcaaaqaamaabmaabaqbaeqabiqaaaqaaiabdEeahbqaaiabd6gaUbaaaiaawIcacaGLPaaaaaaaleaacqWGPbqAcqGH9aqpcqaIWaamaeaacqWGRbWAcqGHsislcqaIXaqma0GaeyyeIuoakiabc6caUiaaxMaacaWLjaWaaeWaaeaacqaIXaqmaiaawIcacaGLPaaaaaa@59AA@

Equation 1 measures whether a pathway specific subset is more enriched in organisms from a particular category than expected by chance.

Conversely, hypergeometric distribution was also applied to model the probability of observing by chance, at most k organisms in a pathway specific subset size n belonging to a category containing C organisms from the total number of G organisms, such that the P_depleted_-value was given by

Pdepleted=∑i=0k(Ci)(G−Cn−i)(Gn).     (2)
 MathType@MTEF@5@5@+=feaafiart1ev1aaatCvAUfKttLearuWrP9MDH5MBPbIqV92AaeXatLxBI9gBaebbnrfifHhDYfgasaacH8akY=wiFfYdH8Gipec8Eeeu0xXdbba9frFj0=OqFfea0dXdd9vqai=hGuQ8kuc9pgc9s8qqaq=dirpe0xb9q8qiLsFr0=vr0=vr0dc8meaabaqaciaacaGaaeqabaqabeGadaaakeaacqWGqbaudaWgaaWcbaGaeeizaqMaeeyzauMaeeiCaaNaeeiBaWMaeeyzauMaeeiDaqNaeeyzauMaeeizaqgabeaakiabg2da9maaqahabaWaaSaaaeaadaqadaqaauaabeqaceaaaeaacqWGdbWqaeaacqWGPbqAaaaacaGLOaGaayzkaaWaaeWaaeaafaqabeGabaaabaGaem4raCKaeyOeI0Iaem4qameabaGaemOBa4MaeyOeI0IaemyAaKgaaaGaayjkaiaawMcaaaqaamaabmaabaqbaeqabiqaaaqaaiabdEeahbqaaiabd6gaUbaaaiaawIcacaGLPaaaaaaaleaacqWGPbqAcqGH9aqpcqaIWaamaeaacqWGRbWAa0GaeyyeIuoakiabc6caUiaaxMaacaWLjaWaaeWaaeaacqaIYaGmaiaawIcacaGLPaaaaaa@55EC@

Equation 2 measures whether a pathway specific subset is more depleted to organisms from a particular category than expected by chance.

### Computing distances between organism-specific pathway graphs

To compute the distances between organism-specific pathway graphs, we used a definition based on the topological relationship between shared vertices. Assume a certain pathway is present in two organisms i and j. If the two organism-specific pathway graphs contain G_i _and G_j _vertices, respectively, with n shared vertices, and each shared vertex has k_i _and k_j _neighbors, then the topological distance between two organism-specific pathway graphs can be defined as

dij=1−∑n2ki∩kjki+kjGi×Gj.     (3)
 MathType@MTEF@5@5@+=feaafiart1ev1aaatCvAUfKttLearuWrP9MDH5MBPbIqV92AaeXatLxBI9gBaebbnrfifHhDYfgasaacH8akY=wiFfYdH8Gipec8Eeeu0xXdbba9frFj0=OqFfea0dXdd9vqai=hGuQ8kuc9pgc9s8qqaq=dirpe0xb9q8qiLsFr0=vr0=vr0dc8meaabaqaciaacaGaaeqabaqabeGadaaakeaacqWGKbazdaWgaaWcbaGaemyAaKMaemOAaOgabeaakiabg2da9iabigdaXiabgkHiTmaalaaabaWaaabuaeaadaWcaaqaaiabikdaYiabdUgaRnaaBaaaleaacqWGPbqAaeqaaOGaeyykICSaem4AaS2aaSbaaSqaaiabdQgaQbqabaaakeaacqWGRbWAdaWgaaWcbaGaemyAaKgabeaakiabgUcaRiabdUgaRnaaBaaaleaacqWGQbGAaeqaaaaaaeaacqWGUbGBaeqaniabggHiLdaakeaadaGcaaqaaiabdEeahnaaBaaaleaacqWGPbqAaeqaaOGaey41aqRaem4raC0aaSbaaSqaaiabdQgaQbqabaaabeaaaaGccqGGUaGlcaWLjaGaaCzcamaabmaabaGaeG4mamdacaGLOaGaayzkaaaaaa@52BF@

As an alternative, we also used a more complex distance definition of Heymans and Singh [5], which is based on the similarity between vertices, measured by the similarity between EC numbers, and the structural relationship between vertices. (Software used for calculating the distance of Heyman and Singh's definition was kindly obtained from A. K. Singh.)

### Building metabolic pathway based phylophenetic trees

Under the definition of presence or absence of pathways, for each metabolic pathway, there was a pathway specific subset of organisms that contained it. After selecting all prokaryotes from the subset, a distance matrix was obtained by computing the distances between all pairs of studied organisms. The program NEIGHBOR in the PHYLIP package with the neighbor-joining algorithm [28] was used to build a metabolic pathway based phylophenetic tree from the distance matrix. For the same set of organisms, random trees were generated with a BioPerl [29] object called Bio::Tree::RandomFactory.

### Building 16S rRNA based trees

Prokaryote 16S rRNA gene sequences were obtained from the Ribosomal Database Project-II [30]. The exception was the 16S rRNA gene of *Pyrobaculum aerophilum*, which contains an intron [31] and for which the sequence was derived by splicing according to the annotation in NCBI. The program CLUSTALW [32] was used to do multiple sequence alignment for all 171 16S rRNA genes, and thereafter the program DNADIST in the PHYLIP package with default settings was used to calculate a distance matrix based on the alignments. Organisms were selected to correspond to the metabolic pathway based trees, distances matrices were derived, and trees were build using the PHYLIP package as above.

### Combining total single pathways based trees into one tree

The quartet method was used to reconstruct a combined tree based on the set of single pathway trees. In order to compare this combined tree to gene content and gene order based trees, we limited tree reconstruction to 47 organisms for which relevant data was also available in the SHOT server (Additional file 1; [14]). For each pathway-specific subset with more than 4 organisms represented in SHOT, a distance matrix was obtained by computing the distances between all pairs of shared organisms, from which the topology of a pathway based tree was inferred. A set of quartets was inferred from the distance matrix by the program Distquart in the PhyloQuart package [33] and all pathway specific quartet files were transformed and combined into one quartet file. By using the Q* method [33], from the PhyloQuart package, a file of bipartitions was obtained. The whole pathway set based tree for the representative set of organisms was inferred by the program Tree-pop in the PhyloQuart package. (No branch length information was provided by this program.)

In addition, two gene content trees and two gene order trees for the 47 organisms were reconstructed using the two versions of the SHOT database, with all settings at default.

### Computing the similarity between trees

The similarity between two trees was computed based on Penny and Hendy's method [34]. One cut at any interior branch divided a tree into two groups; therefore, for an unrooted bifurcating tree with n species, there were n-3 (n>3) cuts resulting in different partitions. No matter being bifurcating trees or multifurcating trees, if tree i and tree j concerned the same set of organisms and there were T_i _and T_j _partitions for tree i and tree j separately, the similarity between them was defined as

s=2Ti∩TjTi+Tj.     (4)
 MathType@MTEF@5@5@+=feaafiart1ev1aaatCvAUfKttLearuWrP9MDH5MBPbIqV92AaeXatLxBI9gBaebbnrfifHhDYfgasaacH8akY=wiFfYdH8Gipec8Eeeu0xXdbba9frFj0=OqFfea0dXdd9vqai=hGuQ8kuc9pgc9s8qqaq=dirpe0xb9q8qiLsFr0=vr0=vr0dc8meaabaqaciaacaGaaeqabaqabeGadaaakeaacqWGZbWCcqGH9aqpdaWcaaqaaiabikdaYiabdsfaunaaBaaaleaacqWGPbqAaeqaaOGaeyykICSaemivaq1aaSbaaSqaaiabdQgaQbqabaaakeaacqWGubavdaWgaaWcbaGaemyAaKgabeaakiabgUcaRiabdsfaunaaBaaaleaacqWGQbGAaeqaaaaakiabc6caUiaaxMaacaWLjaWaaeWaaeaacqaI0aanaiaawIcacaGLPaaaaaa@4256@

Under this definition, the similarity between two trees varies from 0 to 1.

### Robustness tests

Two criterions were used to test the robustness of our approach against different fractions of errors in the underlying data. One criterion added specified errors (from 1% to 10%, with 1% intervals) to the reference graph of each metabolic pathway by randomly altering a specific fraction of edges (removing existing ones or creating new ones). The other method added specified errors (from 1% to 10%, with 1% intervals) to the KEGG annotation of enzymes for each considered organism by randomly choosing a specific fraction of enzymes and then annotating existing enzymes as absent or non-existing enzymes as present. For each interval a metabolic pathway based phylophenetic trees were reconstructed (using the distance definition in equation 3) based on the perturbed pathway graphs or error-added enzyme annotations, and the single pathway based trees were combined into one tree. This process was repeated 100 times, and based on these 100 pathway set based trees a consensus tree with bootstrap-like values was constructed using the program CONSENSE in PHYLIP package.

## Authors' contributions

YZ, SL conceived, designed and performed the study under the supervision of RC; YZ, XZ and BS collected and analyzed the data; SL and ZZ (Zhihua Zhang) wrote the computer code; ZZ (Zefeng Zhang), SS and HL designed algorithms; YZ, GS and RC wrote the manuscript; All authors have read and approved the final manuscript.

## Supplementary Material

Additional File 1 **List of organisms and their categories **The phylogenetic categories of selected organisms were derived from KEGG; the organisms were classified into 3 domains and 27 categories. 47 organisms, the data of which were also available in SHOT server, were marked with *. Click here for file

## Figures and Tables

**Table 1:** List of parameters and similarity values for 37 metabolic pathways. The information on pathway IDs, names and categories was obtained from KEGG [2]. For each pathway reference graph, the diameter and average path length were calculated by regarding the graph as directed, and the clustering coefficient was calculated by regarding the graph as undirected. TopNet [36] was used to calculate the clustering coefficient. The prokaryote number refers to the number of studied prokaryotes containing the pathway, based on the definition of presence or absence of metabolic pathways given in Methods.

pathway ID	pathway name	category	vertex number	diameter	average path length	clustering coefficient	prokaryote number	similarity to 16S rRNA based tree ^a^	similarity to 16S rRNA based tree ^b^
map00010	Glycolysis / Gluconeogenesis	Carbohydrate Metabolism	40	12	4.58	0.617	154	0.178	0.198
map00020	Citrate cycle (TCA cycle)	Carbohydrate Metabolism	23	9	3.06	0.594	118	0.13	0.121
map00030	Pentose phosphate pathway	Carbohydrate Metabolism	34	8	3.24	0.468	107	0.153	0.163
map00040	Pentose and glucuronate interconversions	Carbohydrate Metabolism	53	13	5.28	0.441	14	0.272	0.363
map00051	Fructose and mannose metabolism	Carbohydrate Metabolism	61	10	3.60	0.419	96	0.225	0.182
map00052	Galactose metabolism	Carbohydrate Metabolism	37	14	4.80	0.408	38	0.171	0.142
map00130	Ubiquinone biosynthesis	Metabolism of Cofactors and Vitamins	21	7	3.13	0.088	39	0.222	0.194
map00220	Urea cycle and metabolism of amino groups	Amino Acid Metabolism	33	13	4.46	0.285	110	0.112	0.121
map00230	Purine metabolism	Nucleotide Metabolism	97	16	3.78	0.481	164	0.236	0.204
map00240	Pyrimidine metabolism	Nucleotide Metabolism	59	9	2.95	0.408	170	0.149	0.161
map00251	Glutamate metabolism	Amino Acid Metabolism	35	5	1.83	0.622	148	0.172	0.172
map00252	Alanine and aspartate metabolism	Amino Acid Metabolism	38	7	2.50	0.626	139	0.213	0.198
map00260	Glycine, serine and threonine metabolism	Amino Acid Metabolism	55	14	4.09	0.556	116	0.238	0.212
map00271	Methionine metabolism	Amino Acid Metabolism	23	5	2.43	0.515	122	0.126	0.092
map00280	Valine, leucine and isoleucine degradation	Amino Acid Metabolism	32	9	3.16	0.402	40	0.297	0.270
map00330	Arginine and proline metabolism	Amino Acid Metabolism	70	10	4.14	0.502	70	0.238	0.268
map00340	Histidine metabolism	Amino Acid Metabolism	39	15	5.10	0.276	110	0.056	0.056
map00360	Phenylalanine metabolism	Amino Acid Metabolism	39	4	1.84	0.465	34	0.258	0.258
map00362	Benzoate degradation via hydroxylation	Biodegradation of Xenobiotics	44	9	3.02	0.202	19	0.250	0.187
map00400	Phenylalanine, tyrosine and tryptophan biosynthesis	Amino Acid Metabolism	31	10	3.48	0.464	149	0.075	0.089
map00450	Selenoamino acid metabolism	Metabolism of Other Amino Acids	19	7	2.70	0.253	52	0.142	0.122
map00500	Starch and sucrose metabolism	Carbohydrate Metabolism	74	12	4.23	0.283	82	0.189	0.164
map00520	Nucleotide sugars metabolism	Carbohydrate Metabolism	28	5	2.16	0.548	93	0.044	0.055
map00530	Aminosugars metabolism	Carbohydrate Metabolism	38	7	2.93	0.582	130	0.110	0.133
map00561	Glycerolipid metabolism	Lipid Metabolism	80	13	4.02	0.343	74	0.295	0.211
map00620	Pyruvate metabolism	Carbohydrate Metabolism	67	7	2.43	0.598	155	0.296	0.263
map00630	Glyoxylate and dicarboxylate metabolism	Carbohydrate Metabolism	58	8	3.10	0.618	57	0.129	0.166
map00640	Propanoate metabolism	Carbohydrate Metabolism	45	10	3.05	0.513	21	0.055	0.055
map00650	Butanoate metabolism	Carbohydrate Metabolism	52	13	3.90	0.429	32	0.275	0.275
map00670	One carbon pool by folate	Metabolism of Cofactors and Vitamins	24	4	1.57	0.80	157	0.103	0.116
map00710	Carbon fixation	Energy Metabolism	23	8	3.28	0.247	97	0.117	0.117
map00730	Thiamine metabolism	Metabolism of Cofactors and Vitamins	14	6	2.88	0.199	20	0.117	0.117
map00760	Nicotinate and nicotinamide metabolism	Metabolism of Cofactors and Vitamins	32	8	2.47	0.518	121	0.118	0.118
map00770	Pantothenate and CoA biosynthesis	Metabolism of Cofactors and Vitamins	27	10	3.43	0.320	92	0.134	0.157
map00790	Folate biosynthesis	Metabolism of Cofactors and Vitamins	25	7	2.69	0.322	124	0.115	0.115
map00860	Porphyrin and chlorophyll metabolism	Metabolism of Cofactors and Vitamins	60	20	6.37	0.171	90	0.241	0.183
map00910	Nitrogen metabolism	Energy Metabolism	63	5	1.87	0.508	137	0.231	0.186

^a ^Pathway-based phylophenetic trees were derived by using distance definition in equation 3.

^b ^Pathway-based phylophenetic trees were derived by using Heymans and Singh's distance definition [5].

**Table 2:** Similarities values from comparisons between pathway set based tree and trees based on other information. **The similarity values range from 0 to 1 (1 being identical topology).**

	Pathway set based tree	Gene content tree (SHOT vs. 1.0)	Gene order tree (SHOT vs. 1.0)	Gene content tree (SHOT vs. 2.0)	Gene order tree (SHOT vs. 2.0)	16S rRNA based tree
Pathway set based tree	-	0.409	0.318	0.409	0.295	0.386
Gene content tree (SHOT vs. 1.0)		-	0.613	0.727	0.409	0.545
Gene order tree (SHOT vs. 1.0)			-	0.568	0.363	0.522
Gene content tree (SHOT vs. 2.0)				-	0.477	0.613
Gene order tree (SHOT vs. 2.0)					-	0.340
16S rRNA based tree						-
